# Synthesis, Characterization, and Polishing Properties of a Lanthanum Cerium Fluoride Abrasive

**DOI:** 10.3390/ma16093393

**Published:** 2023-04-26

**Authors:** Yan Mei, Wenjuan Chen, Xuean Chen

**Affiliations:** 1 Faculty of Materials and Manufacturing, Beijing University of Technology, Beijing 100124, China; 2 Key Laboratory of Advanced Functional Materials, Ministry of Education of China, Faculty of Materials and Manufacturing, Beijing University of Technology, Beijing 100124, China

**Keywords:** abrasive, chemical mechanical polishing, ceria, X-ray diffraction

## Abstract

One kind of lanthanum cerium fluoride abrasive was prepared using the raw materials Ce_2_(CO_3_)_3_, La·Ce(CO_3_)_3_, and NH_4_F at temperatures of 400–1050 °C. The combined techniques of X-ray diffraction with Rietveld refinements, scanning electron microscopy, and X-ray photoelectron spectroscopy were employed to characterize the products. It was found that the materials are all made up of agglomerated irregular block-shaped particles with particle sizes in micrometer ranges. Below 850 °C, the product is a mixture of cubic CeO_2_ and trigonal LaF_3_, while above 900 °C, it is a mixture of cubic CeO_2_ and tetragonal LaOF. A higher calcination temperature suppresses the formation of the LaF_3_ phase but enhances the LaOF phase. The Ce in the prepared material is present in mixed states of Ce^3+^ and Ce^4+^, and the Ce^4+^/Ce^3+^ ratio increases with increasing calcination temperature. When the material prepared at 900 °C was used in the polishing test on K9 glass, the obtained polishing surface is very clean and flat, and the thickness difference before and after grinding is moderate, indicating its potential as an abrasive for polishing the surface of optical glass.

## 1. Introduction

Ceria (CeO_2_) is an important rare earth functional material, which is widely used in solid oxide fuel cells, oxygen sensors, catalysts, ultraviolet absorbents and filters, luminescent materials, and polishing media in micro-electronics [1,2,3,4,5,6,7]. In modern manufacturing, the glass substrates’ global planarity is usually achieved by using chemical mechanical polishing (CMP) technology [8]. As one of the most widely used CMP abrasives, CeO_2_ is considered the best abrasive for glass polishing due to its unique chemical reaction with silicon oxide (SiO_2_) [9]. Under the combined action of mechanical wear and chemical reaction, CeO_2_ abrasives have a high material removal rate (MRR) and superior surface finishes. However, due to its high surface activity, easy agglomeration, and regular particle morphology, the use of CeO_2_ results in difficulties in post-CMP cleaning, low efficiency, and precision, which limits its direct use in the precision polishing of glass surfaces. In order to improve the overall cutting effect of the abrasives, many studies have been carried out in controlling the particle size and distribution as well as morphology and surface features [10,11,12]. Several researchers prepared ceria abrasives by annealing carbonate or oxalate precursors or by calcining octahedral CeO_2_ precursors self-assembled from spherical primary nanocrystals and found that the calcination temperature is also a critical parameter that governs the polishing efficiency and the quality of the polished surface [13,14]. In addition to these, Cheng et al. attempted to dope a certain amount of lanthanide elements (La, Nd, and Yb) into cerium dioxide using the modified incipient impregnation method [15]. In this process, trivalent dopants, mainly the rare earth elements, could substitute Ce^4+^, release more oxygen vacancies, and cause partial reduction of neighboring Ce^4+^ to Ce^3+^. Thus, the MRR of the doped CeO_2_ abrasives can be increased by about 30%, simultaneously obtaining good surface quality. Ma et al. prepared a series of Ce_1−x_La_x_O_2_ (x = 0, 0.1, 0.2, 0.3) abrasives by the hydrothermal method [16]. It is found that with the increase in the La^3+^ doping level, the morphology of CeO_2_ particles changes from sphere to octahedron, and the Ce^3+^ concentration on the surface of CeO_2_ abrasives first increases significantly and then tends to saturate. The MRR of the abrasives shows a similar trend with the Ce^3+^ surface concentration.

What is more, most commercially available polishing powders are fluorinated, because the fluorine (F) atoms can significantly improve the chemical activity of ceria in the polishing process, change the particle morphology and reduce agglomeration between particles [17]. The fluorination of La^3+^-doped CeO_2_ can form two additional phases, namely LaOF and LaF_3_, of which LaOF has a similar polishing ability to CeO_2_, while LaF_3_ with low hardness is easily crushed during polishing, which lowers the MRR and shortens the useful life of ceria-based abrasives [18]. For the CeO_2_-LaOF-based abrasives, only three studies are available in the literature, i.e., Pei et al. prepared the ceria-based compounds with additions of La and F (CLF compounds) by using industrial-grade fluorinated lanthanum cerium carbonate as a precursor via a facile calcination method. Subsequently, the evolution of phase structures of the compounds during preparation and the relationship between the structure and the polishing performance were investigated [18]. Zheng et al. used NH_4_HCO_3_ and (Ce_0.7_, La_0.3_)_2_(SO_4_)_3_·xH_2_O as raw materials to synthesize rare earth carbonate precursors by parallel feeding in precipitation. Then, ceria-based abrasives were obtained by fluorating and calcinating rare earth carbonates of different shapes, and the effects of precursors on the morphology and CMP performance of ceria-based abrasives were studied [19]. Zhao et al. developed a novel composite abrasive polishing slurry, which consists of cerium oxide (CeO_2_), lanthanum oxyfluoride (LaOF), potassium pyrophosphate (K_4_P_2_O_7_), sodium N-lauroyl sarcosinate (SNLS), and sodium polyacrylate (PAAS). Through orthogonal experiments, the optimal polishing parameters and slurry composition ratios were obtained, and the CMP mechanism was analyzed [20].

With the development of the precision optical industry and the emergence of new applications, the requirements for glass surface finish are becoming increasingly high. Therefore, it is very important and necessary to develop new abrasives with higher removal rates. Although some studies have been conducted on the ceria-based compounds with additions of La and F as mentioned above, research on the relationship between the structure and polishing performance of this system is still limited. In this work, a new lanthanum cerium fluoride abrasive was reported, which contains Ce^4+^/Ce^3+^, La^3+^, and F^−^ ions, so that such a slurry is expected to maintain the high chemical activity of ceria (a typical abrasive CMP slurry). The synthesis, phase composition, morphology, XPS, and polishing properties of this new abrasive were presented. In particular, the phase composition of this system obtained from Rietveld refinements has not been reported in the literature. We believe that this work will be helpful for understanding the structure–property relationship of cerium-based abrasives and contribute to the development of new abrasives for polishing optical glass surfaces.

## 2. Materials and Methods

### 2.1. Materials Synthesis

All reagents are purchased from Shandong Jingze Optical Material Co., Ltd. (Shandong, China) and have not been further purified before use. In a typical synthesis, a mixture of 0.834 g NH_4_F (T. P.), 8.00 g Ce_2_(CO_3_)_3_ (T. P.), and 2.00 g La·Ce(CO_3_)_3_ (T. P.) in the molar ratio of 5.2:4:1 was mixed thoroughly and a certain among of distilled water was added to adjust the mixture into slurry. After that, drying at 200 °C for 4 h was implemented, and the product was transferred to a corundum crucible, which was subsequently placed in a muffle oven and heated at a rate of 5 °C/min to the target temperature (e.g., 400 °C, 500 °C, 600 °C, 700 °C, 750 °C, 800 °C, 850 °C, 900 °C, 950 °C, 1000 °C, and 1050 °C) and kept at this temperature for 3 h. Afterward, the furnace was switched off, and the sample was naturally cooled to room temperature and collected for further characterization.

### 2.2. Materials Characterization

The chemical compositions of the sample were measured on a Perkin Elmer Optima 7000 DV inductively coupled plasma optical emission spectrometer (ICP-OES, Waltham, MA, USA). Powder X-ray diffraction (XRD) data were collected at ambient temperature in a continuous mode using the monochromatized Cu K_α1_ radiation (*λ* = 1.5406 Å) of a Bruker D8 ADVANCE (Bruker AXS) diffractometer operating at 40 kV and 40 mA. Data were collected in the 2θ range of 10°–80° for the phase identification (5°–100° for Rietveld refinements) with a step size of 0.02° and a step time of 10 s. The average crystallite size of the product was estimated based on the full width at half-maximum (FWHM) of the CeO_2_ (111) peak using Scherrer’s equation: D = 0.89*λ*/(*β*cos*θ*), where *λ* = 1.54056 Å is the wavelength of the X-ray radiation, *β* and *θ* are FWHM and the Bragg angle, respectively. The surface morphology of the product was investigated using a field emission scanning electron microscope (FEG-SEM, Quanta 450 FEG, FEI, Hillsboro, OR, USA) with an acceleration voltage of 5.0 kV. X-ray photoelectron spectroscopy (XPS) measurements were carried out on an ESCALAB 250xi X-ray photoelectron spectrometer (Thermo Fisher Scientific, Waltham, MA, USA) of Al K_α_ X-ray radiation (1486.6 eV), with the binding energy calibrated by the C1s peak (284.6 eV). The polishing test was carried out using an intelligent precision system of super polishing machine (NF-300, NANO FACTOR, Tokyo, Japan). The scratch on the surface of the glass workpiece was evaluated using a surface defect detector made in Japan, where a lamp of 1300~1500 lumens was used as the light source. The surface quality of glass was checked with a magnifier (with a 40 × objective) made by Shandong Jingze Company, Shandong, China. The thickness difference of the glass workpiece per unit of time before and after grinding was measured via a micrometer screw with an accuracy of 0.001 mm. Five repeated experiments were conducted, and the average thickness difference was used to plot the graph.

## 3. Results and Discussion

### 3.1. XRD Analysis

In the temperature range of 400–1050 °C, the percentage composition (the average value, wt.%) of the calcined products was determined by ICP-OES to be CeO_2_~92.56%, La_2_O_3_~7.44%, and the F content was determined by fluoride-ion electrode method to be ~3.62%. The color of the product becomes lighter with the increase in the calcination temperature, which is yellow, light yellow, yellow–white, and white, respectively.

**Figure 1:**
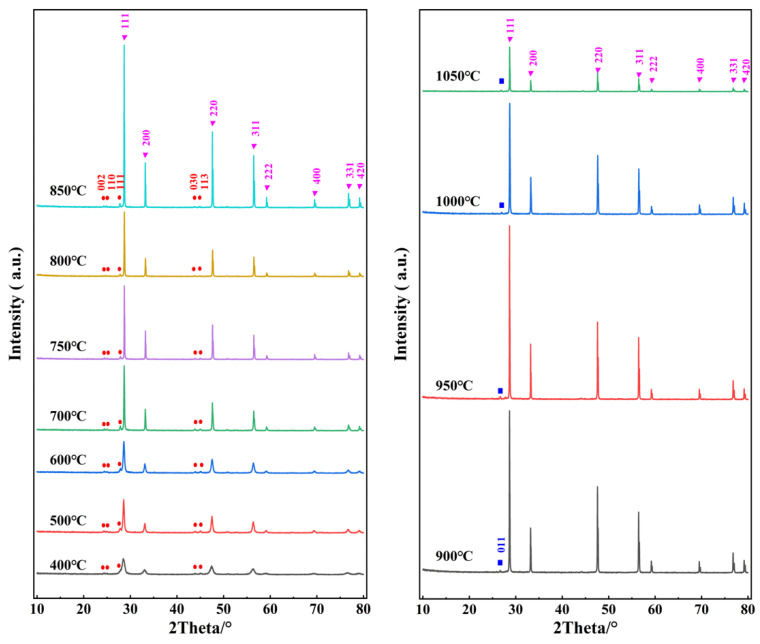
XRD patterns of the cerium-based abrasive calcined at 400–1050 °C. ▼: CeO_2_ (PDF 34-0394), ●: LaF_3_ (PDF 84-0942), ■: LaOF (PDF 89-5168).

Figure 1 shows the temperature-dependent XRD patterns of the as-prepared cerium-based abrasive calcined at 400–1050 °C. Table 1 lists the corresponding XRD parameters, including the 2θ positions of the CeO_2_ (111), LaF_3_ (111), and LaOF (011) main peaks, as well as the lattice constant calculated from the XRD spectra, the average grain size estimated by Scherrer formula, and the intensity percentage ratio of LaF_3_ (111) or LaOF (011) to CeO_2_ (111), etc. Obviously, the main phase of all calcined samples is the cubic fluorite structure of CeO_2_, which has XRD peaks corresponding to planes (111), (200), (220), (311), (222), (400), (331), and (420). At the calcination temperatures of 400–850 °C, the product is mainly composed of cubic cerium dioxide (PDF 34-0394) mixed with a small amount of trigonal lanthanum fluoride (PDF 84-0942). The main diffraction peak of cubic CeO_2_ is located at about 2θ = 28.5~28.7°, while that of the trigonal LaF_3_ appears at 2θ = 27.7~27.9°. With the increasing calcination temperature from 400 to 850 °C, the intensity percentage ratio of the main diffraction peak of LaF_3_ to that of CeO_2_ decreases successively from 14.2% to 1.9%, reflecting the fact that the thermal stability of LaF_3_ becomes worse with increasing temperature. Within this temperature range, FWHM generally decreases and the average crystallite size increases accordingly, indicating that the particle grows with increasing calcination temperature.

It is worth noting that the phase appearing in the product is LaF_3_ instead of CeF_3_ and La_2_O_3_. Although LaF_3_ is isostructural with CeF_3_, the outermost electron configuration of the elements La and Ce is different (5d^1^6s^2^ vs. 4f^1^5d^1^6s^2^), and the ionic radius of La^3+^ is larger than that of Ce^3+^ (R_La3+_ = 1.061 Å vs. R_Ce3+_ = 1.034 Å), consequently, La^3+^ has a more metallic character and can form compounds with F^−^ more easily than Ce^3+^ ion. In addition, from an electronegativity point of view, metallic elements with smaller electronegativity and non-metallic elements with larger electronegativity are more active [21,22]. The greater the difference between the electronegativity of metallic and non-metallic elements, the more stable their ionic bonded compounds will be. LaF_3_, CeF_3_, and La_2_O_3_ are all ionic bonded compounds, considering the electronegativity of La (1.10), Ce (1.12), O (3.44), and F (3.98), it is reasonable to believe that LaF_3_ is more stable than CeF_3_ and La_2_O_3_.

With the further rise of calcination temperature to 900–1050 °C, the product primarily consists of cubic cerium dioxide, with small admixtures of tetragonal lanthanum oxide fluoride (PDF 89-5168). The main diffraction peak (011) of the tetragonal LaOF is at 2θ = 26.7~27.0°, and the intensity percentage ratio of the main diffraction peak of LaOF to that of CeO_2_ is 1.5%~4.5%. It is clear from Figure 1 that the LaF_3_ phase was found in the samples obtained at lower temperatures, but no longer exists at temperatures above 900 °C. Overall speaking, above 900 °C, the particle size decreases slightly and the intensity ratio of LaOF (011)/CeO_2_ (111) increases with rising temperature, indicating that a higher calcination temperature leads to the partial melting of the sample and enhancement of the LaOF phase. Some studies have shown that LaOF has a similar grinding ability to CeO_2_ [18]. It can realize the effective combination of chemical and mechanical action, thus improving the overall cutting speed and polishing effect of abrasives. Therefore, the mixture of CeO_2_ and LaOF obtained at 900–1050 °C is expected to be beneficial for polishing.

From Table 1, we can also find that the lattice parameter *a* of CeO_2_ at 400–500 °C (5.412(2)–5.413(1) Å) is very close to that reported for CeO_2_ in the standard data (5.4113 Å, PDF 34-0394), but it is slightly reduced at 600–1050 °C. A similar situation was observed in some previously reported CeO_2_ samples, which is related to the fact that the lattice parameters of nanocrystalline powders vary with the particle size, and can be explained by the grain-surface relaxation model [23,24]. In addition, as seen in Figure 1, when the calcination temperature is increased, the reflection peaks become narrower, and some of them are split, reflecting the improvement in crystallinity. Below 850 °C, the diffraction intensity generally increases with temperature, while above 1000 °C, it is remarkably weakened. Based on the above analysis, we can conclude that well-crystallized samples can be obtained between 850 and 950 °C. The FE-SEM micrographs to be discussed in the next section support this point.

**Figure 2:**
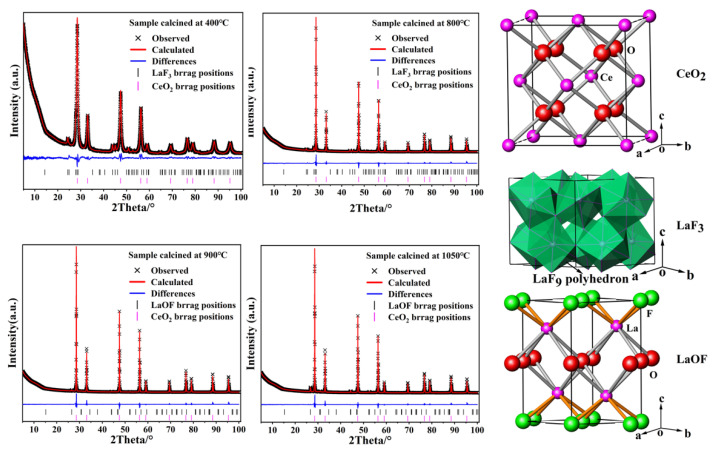
Rietveld refinements of powder XRD files of the cerium-based abrasive calcined at 400, 800, 900, and 1050 °C, as well as the crystal structures of CeO_2_, LaF_3_, and LaOF.

It is well known that the Rietveld refinement method can be used to quantify the phase compositions of a multiphase sample. This method allows the generation of a calculated XRD profile for each phase in the multiphase mixture from its known crystal structure. Subsequently, all calculated patterns are fitted to the observed XRD profile of a multiphase sample by iterative least-squares analysis to find the optimal individual phase scales. The phase scales are then used to determine the percentage of the different phases (phase fractions) of the sample. In order to further understand the crystal structure and phase compositions of the as-synthesized cerium-based abrasive, Rietveld refinements were performed on the powder XRD data using the TOPAS software package [25]. In the refining process, the modified Thompson–Cox–Hastings pseudo-Voigt function [26] was used to simulate the individual XRD peak profile, and the initial structural models were obtained from the Inorganic Crystal Structure Database (ICSD#621716 for CeO_2_, ICSD#201865 for LaF_3_, and ICSD#76427 for LaOF) [27,28,29]. The atomic occupancies and equivalent isotropic displacement parameters remain unchanged, while the lattice parameters, positional coordinates, phase fraction, polynomial background, profile shape, and specimen displacement parameters were refined simultaneously. The observed and calculated XRD patterns as well as their differences and the Bragg positions of four representative samples are shown in Figure 2 and the detailed crystallographic data extracted from the refinements together with fitting parameters are summarized in Table 2. Apparently, the difference between XRD patterns of the experimental and calculated data display small differences in the scale of intensity, the agreement indices R_wp_ are all smaller than 6%, and GOF (goodness of fitting) indicators are less than 4, indicating a good quality of the fitting. The refinement results further confirmed that CeO_2_, LaF_3_, and LaOF in the studied samples crystallize in the cubic *Fm*-3*m*, trigonal *P*-3*c*1, and tetragonal *P*4/*nmm* space group, respectively, with lattice constants in good accord with those reported in the literature [27,28,29]. Below 800 °C, the product is a mixture of cubic CeO_2_ and trigonal LaF_3_, while above 900 °C, it is a mixture of cubic CeO_2_ and tetragonal LaOF. As the temperature increases, the phase fraction of LaF_3_ decreases, while that of LaOF increases, which is highly consistent with our previous argument.

Figure 2 also illustrates the crystal structures of CeO_2_, LaF_3_, and LaOF. It can be seen that CeO_2_ possesses a fluorite-type cubic structure [space group *Fm*-3*m*, with the Ce atoms at the Wyckoff 4*a* and O atoms at the 8*c* sites], which can be described as cations occupying alternate cube centers within a simple cubic array of anions or as anions occupying all the tetrahedral interstices within a face-centered cubic (*f.c.c.*) array of cations. LaF_3_ exhibits the tysonite structure that belongs to the trigonal *P*-3*c*1 space group with six formula units in the unit cell. The crystal structure may be viewed as a hexagonal closed-packed lanthanum sublattice, with fluorine ions occupying interstitial positions. Furthermore, lanthanide ions occupy the Wyckoff 6*f* positions, and the three non-equivalent fluorine ions are placed at 2*a*, 4*d*, and 12*g* sites. LaOF crystallizes in the tetragonal *P*4/*nmm* space group, in which each La^3+^ ion occupies the site of C_4v_ symmetry and is coordinated by four O^2−^ and four F^−^ anions, while both O^2−^ and F^−^ ions reside in the sites of D_2d_ symmetry and are tetrahedrally coordinated to La^3+^ ions. In this structure, (O)_n_*^2n^*^−^ and (F)_n_*^n^*^−^ anionic layers are alternately stacked along the crystallographic *c*-axis direction, and La^3+^ cations are placed in the square prismatic cavities between the layers to hold them together via La-O and La-F bonds, thus obtaining a 3D (LaOF)_n_ framework.

### 3.2. Sample Morphology

**Figure 3:**
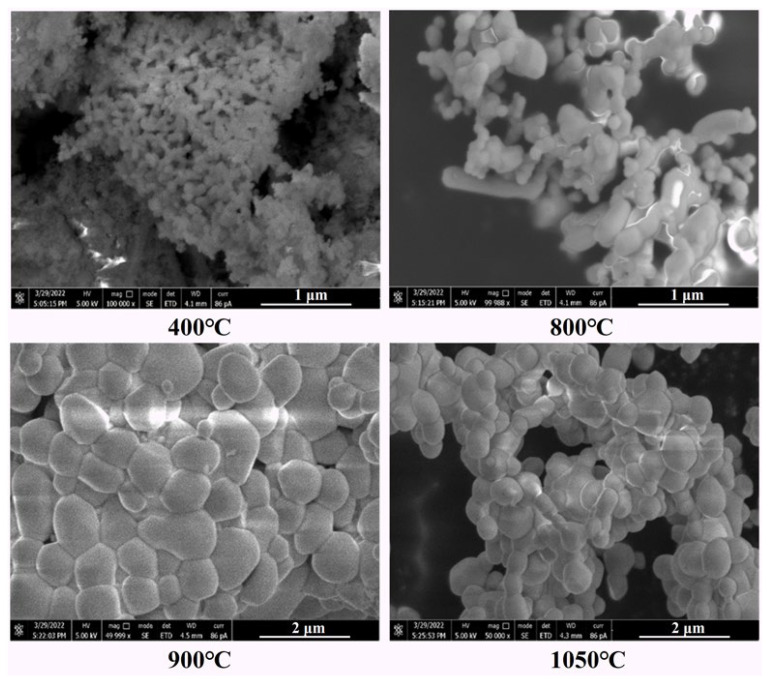
FE-SEM images of the cerium-based abrasive calcined at 400, 800, 900, and 1050 °C.

For the SEM measurements, the products calcined at 400 °C, 800 °C, 900 °C, and 1050 °C were first placed on the sample base via the conductive adhesive, which was subsequently transferred to the vacuum chamber of the Quanta 450 FEG system. After vacuuming, the morphological characteristics of the samples were investigated. In order to protect the samples and ensure the accuracy of element analyses, the samples were not sprayed with gold before the test. Figure 3 shows the representative SEM images of the as-synthesized polycrystalline samples. Obviously, the materials calcined in the temperature range of 400–1050 °C are all made up of agglomerated irregular block-shaped particles with particle sizes in micrometer ranges. Such morphology has the typical inherent characteristics of the adopted high-temperature solid-phase reaction. When calcined at 400 °C, the product presents a loose pore state, which is probably caused by the decomposition of ammonia fluoride and rare earth carbonates accompanied by the release of gas during the low-temperature calcination. As the temperature increases, the particle sizes become larger and larger. When the calcination temperature reaches 900 °C, the obtained products have uniform particle size and good morphology. Once the calcination temperature is further raised to 1050 °C, the products show partial fusion, and thus, the agglomeration between grains is more obvious. All these observations are highly consistent with the aforementioned XRD results, i.e., the increase in calcination temperature leads to the enhancement of crystallinity and grain size.

### 3.3. XPS Spectra

**Figure 4:**
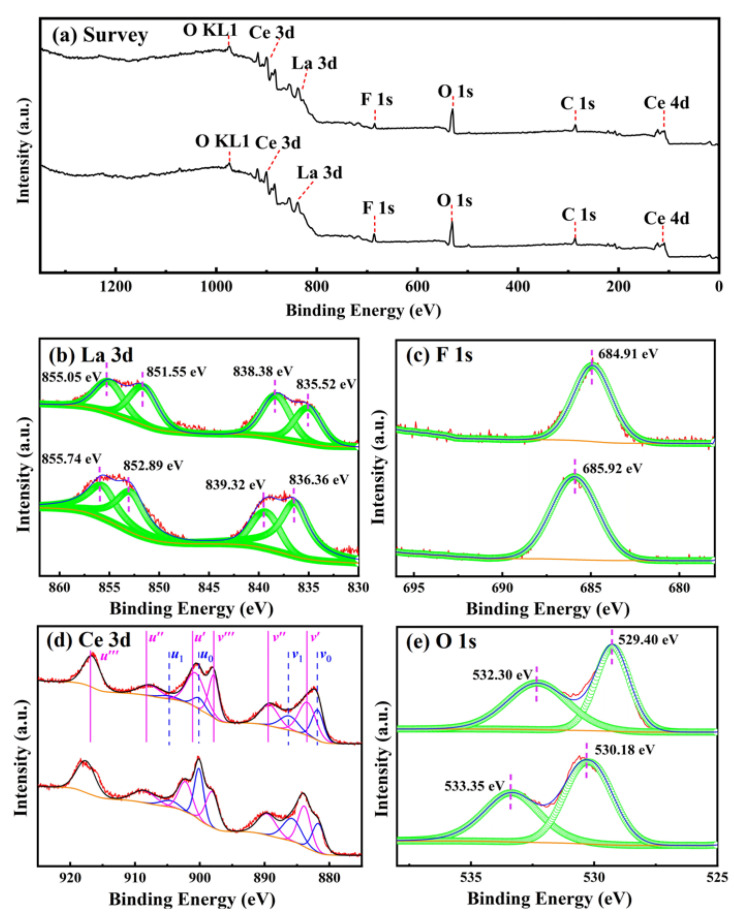
Survey (**a**) and core-level spectra of La 3d (**b**), F 1s (**c**), Ce 3d (**d**), and O 1s (**e**) for the samples prepared at 800 (**bottom**) and 900 °C (**top**).

XPS measurements were performed to study the surface composition and oxidation states in the cerium-based abrasives calcined at 800 and 900 °C. Figure 4a presents the wide survey spectra recorded in the range of 1350–0 eV, which contains Ce 3d, Ce 4d, La 3d, F 1s, O 1s, and C 1s core levels along with O Auger peaks in both samples. No other impurity elements are detected, except for adventitious carbon from the XPS instrument itself. In order to obtain detailed information on the oxidation state of the surface elements, the high-resolution XPS spectra of La 3d, F1s, Ce 3d, and O 1s were recorded, and the results are shown in Figure 4b–e, respectively. Clearly, the La 3d spectrum was fitted such that the two components representing La 3d_3/2_ are located at 855.74 (or 855.05) and 852.89 (or 851.55) eV, whereas the components due to La 3d_5/2_ are located at 839.32 (or 838.38) and 836.36 (or 835.52) eV, thereby confirming the presence of La^3+^ ions in the 800 (or 900 °C) sample [30]. The two components of La 3d_5/2_ and La 3d_3/2_ result from spin–orbit interactions that occur separately in each spin–orbit component [31]. As for the F 1s core level spectrum, it shows a symmetric single peak with binding energies (BEs) of 685.92 and 684.91 eV for the samples prepared at 800 and 900 °C, respectively, which are typical values in the range observed in F^−^-containing compounds [32,33]. In the case of ceria, orbital splitting and a series of energy transfer processes between electrons create a total of ten photoelectron peaks in the Ce 3d spectrum, which are labeled as *u* and *v* for the 3d_3/2_ and 3d_5/2_ contributions, respectively [14,34]. Among them, two doublets (*u*_0_, *v*_0_) and (*u*_1_, *v*_1_) are indexed to two different final states of Ce^3+^ in CeO_2_ owing to the emission from the spin–orbit split 3d_3/2_ and 3d_5/2_ core levels, while the rest of three doublets (*u*′, *v*′), (*u*″, *v*″), and (*u*‴, *v*‴) result from different final states of Ce^4+^. The concentration of Ce^3+^ ions on the surfaces of the abrasives was estimated by dividing the integrated area corresponding to Ce^3+^ by the entire integrated area of Ce 3d, as shown in Table 3. It is clear that the Ce^3+^ concentration decreases from 25.13% to 22.73% with increasing calcination temperature from 800 to 900 °C. In addition, the O 1s spectra can be resolved into two components, indicating the presence of different oxygen species in the sample. The BE peaks located at 530.18 (or 529.40) eV are associated with the lattice oxygen, while those centered at 533.35 (or 532.30) eV can be attributed the O–H bond, adsorbed oxygen, and oxygen defects [35,36,37]. Some studies have shown that the conversion of Ce^3+^ to Ce^4+^ in CeO_2_ easily led to the shift of oxygen binding energy to a low position [14,38,39]. In the present case, the BE value for the characteristic peak of lattice oxygen is shifted from 530.18 to 529.40 eV, as the temperature varies from 800 to 900 °C. This means that the Ce^4+^ content increases with the increment of calcination temperature, which is in accordance with the Ce 3d XPS analysis. Based on the above observations, we can conclude that cerium in the cerium-based abrasives shows the mixed valance states of both Ce^3+^ and Ce^4+^ due to the highly nonstoichiometric nature of ceria, and the Ce^4+^/Ce^3+^ ratio increases with increasing calcination temperature. A similar situation has also been observed by Li et al. when they calcined the hierarchical CeO_2_ octahedrons at different temperatures [14]. Many investigations have shown that the surface Ce oxidation state (Ce^4+^/Ce^3+^) has a significant influence on the polishing performance of CeO_2_ particles. It was reported that in an aqueous medium, Ce^3+^ (rather than Ce^4+^) ions act as active sites for H_2_O dissociation and help the formation of Ce–OH groups at the CeO_2_ surface. Silicate ions can adsorb onto the CeO_2_ surface through interaction with the −OH groups to form the Ce-O-Si bonds during polishing [14,15,40]. In the present case, the cerium-based abrasive prepared at the extremely high temperature (e.g., 1050 °C) may have a lower surface Ce^3+^ concentration, which corresponds to a decrease in the number of active sites on the particle surface and is detrimental to the decomposition of Si–O bonds and the formation of Ce-O-Si bonds in the glass CMP process, and thus, poor surface quality is expected to be observed for K9 glass with this abrasive. The polishing experiments we will discuss in the next section have confirmed this.

### 3.4. Assessment of Polishing Performance

**Figure 5:**
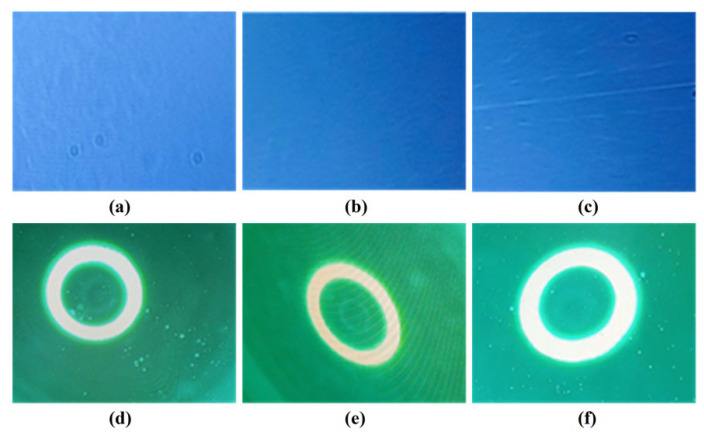
The observed scratches on the K9 glass surface (**top**) and the surface quality of the glass (**bottom**) when using the abrasives prepared at 800 °C (**a**,**d**), 900 °C (**b**,**e**), and 1050 °C (**c**,**f**).

The abrasives prepared at 800, 900, and 1050 °C were added a certain among of pure water to obtain their respective grinding slurries, which have a solid content of 100 g/L. With these slurries, a polishing test was performed on the K9 glass workpiece for 60 min by using the NF-300 system. Subsequently, the polished workpiece is cleaned with ultra-pure water followed by drying. A surface defect detector was used to evaluate the scratches on the glass surface based on the reflection method under the dust-free condition. For this measurement, the scratches are counted in such a way that 100 is the full score for the sample without scratching. The greater the scratch counts, the better the polishing effect. As shown in Figure 5a–c, the scratch counts on the glass surface were determined to be 89, 95, and 57 when using the cerium-based abrasives calcined at 800, 900, and 1050 °C, respectively. Thus, the product prepared at 900 °C displays the best polishing effect.

The surface quality and adhesion state of the K9 glass were checked by a magnifier equipped with a 40× lens, as shown in Figure 5d–f. It can be seen that when the K9 glass surface was polished by using the abrasive prepared at 800 °C, serious white spots, and some adherents were observed on the polished surface. This may be due to the fact that a low calcination temperature leads to a large specific surface area and a high reaction activity of the prepared abrasive. In the grinding process, such an abrasive has strong chemical adsorption with the glass surface, which makes the calcined particles adhere to the glass surface and difficult to be cleaned. If the abrasive prepared at 900 °C was used, the obtained polishing surface is very clean and flat. Furthermore, when the product prepared at 1050 °C was used to polish the K9 glass, there are some pits on the polished surface, which means that with a further increase in the calcination temperature, the polishing powder is completely crystallized, the crystalline defects are significantly reduced, and thus, the mechanical hardness of the abrasive particles are remarkably improved. During the polishing process, the abrasive particles may damage the glass surface under the action of mechanical friction, resulting in the formation of pits. Therefore, the smoothness of the polished surface becomes poor. According to the mechanism proposed by Cook [41,42], when CeO_2_ is dispersed in water, it contains Ce-OH groups, which react with the Si-O^−^ sites on the glass surface to form Ce-O-Si bonds, and then release Si(OH)_4_ into the solution. The proposed reaction is written as: -Ce-OH + -Si-O^−^ ↔ -Ce-O-Si- + OH^−^. Because the Ce-O-Si bonds are stronger than Si-O-Si bonds, this surface layer activated by the chemical reaction can be removed by the mechanical friction of the abrasive particles. Therefore, the polishing of the silica surface is the result of the combined action of both chemical reaction and mechanical friction. When the calcination temperature is 800 °C, the chemical action of the abrasive particles is stronger than the mechanical one, and thus, serious white spots and some adherents were observed on the polished surface. At the extremely high calcination temperature of 1050 °C, the chemical effect is weak, and the mechanical action is dominant, resulting in some pits on the polished surface. The material calcinated at 900 °C shows low surface roughness and the best surface quality for K9 glass, indicating that it can achieve the best match between chemical activity and mechanical hardness of the particles.

**Figure 6:**
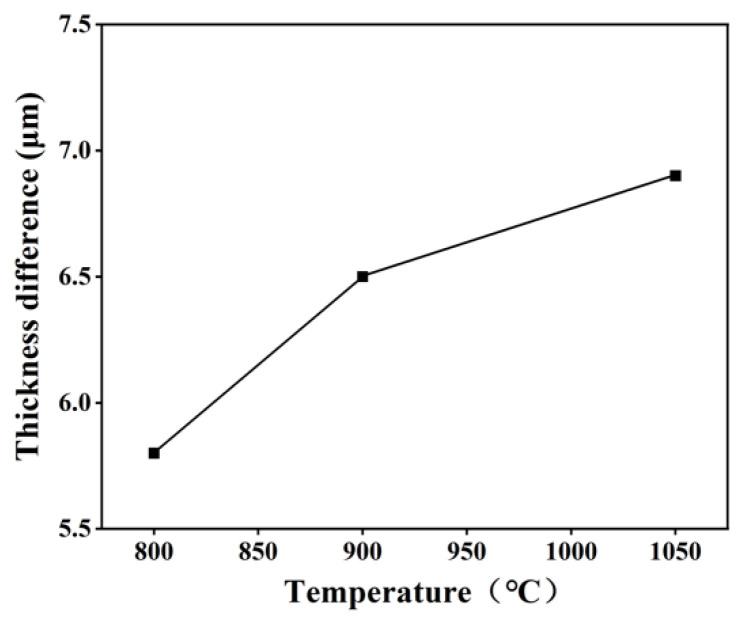
The thickness difference of the glass workpiece before and after grinding with the abrasive prepared at different temperatures.

Figure 6 shows the thickness difference of the glass workpiece per unit of time before and after grinding when using the abrasives prepared at different temperatures. Obviously, with the calcination temperature increasing from 800 to 1050 °C, the thickness difference of the glass workpiece first increases rapidly and then increases slowly. As mentioned above, the increase in calcination temperature leads to an increase in grain size and crystallinity. Under the same polishing conditions, the larger the primary particle size of the polishing powder, the sharper the edges and corners of the particles, and the higher the material removal rate. This explains why the thickness difference of the glass workpiece increases rapidly when the calcination temperature increases from 800 to 900 °C. However, once the calcination temperature is further raised beyond 900 °C, the chemical effect weakens, resulting in a slow increase in the thickness difference. In combination with the number of scratches produced during grinding, the surface quality of the glass after grinding, and the thickness difference before and after grinding, we can conclude that the material calcinated at 900 °C displays the best surface quality and moderate thickness difference for K9 glass.

It is noteworthy that, in previous work [18], the fluorinated lanthanum cerium carbonate precursor was first synthesized using industrial-grade cerium lanthanum carbonate, HF (30%) solution, and NH_4_HCO_3_ as raw materials. Then, the CLF compound polishing powders were obtained by calcinating the fluorinated rare earth carbonates. It was found that the compounds are composed of three phases: CeO_2_, LaOF, and LaF_3_, and a higher degree of fluorination and a higher calcination temperature led to the formation of more LaOF and fewer LaF_3_ phases. The LaF_3_ phase deteriorates, while the LaOF phase improves polishing performance. Our current work has confirmed their viewpoint. In addition, based on XPS analyses and Rietveld refinements of powder XRD patterns, we also provided the Ce^3+^ concentration and phase compositions of the CLF compounds. These studies were not conducted in the aforementioned work.

## 4. Conclusions

In this work, one kind of lanthanum cerium fluoride abrasive was prepared at 400–1050 °C. XRD analyses combined with Rietveld refinements show that at low calcination temperatures, the product is mainly composed of cubic CeO_2_ mixed with a small amount of trigonal LaF_3_, while at high temperatures, it primarily consists of cubic CeO_2_, with small admixtures of tetragonal LaOF. With the increasing calcination temperature from 400 to 800 °C, the phase fraction of LaF_3_ decreases from 9.76 (18)% to 6.95 (19)%, while as the temperature further increases from 900 to 1050 °C, the phase fraction of LaOF increases from 1.39 (18)% to 4.53 (15)%. The SEM images reveal that the increase in calcination temperature leads to the enhancement of crystallinity and grain size, and the product obtained at 900 °C has uniform particle size and good morphology. The XPS studies indicate the existence of La^3+^, O^2−^, and F^−^ as well as the mixed states of Ce^3+^ and Ce^4+^ in the sample. With the increase in calcination temperature from 800 to 900 °C, the Ce^3+^ concentration decreases from 25.13% to 22.73%, which is unfavorable for the chemical reaction of Ce^3+^ ions with the glass surface to achieve material removal. The polishing experiments revealed that the material calcinated at 900 °C displayed the best surface quality for K9 glass because of the best matching of chemical activity and mechanical hardness. This material may serve as a potential abrasive in the area of glass polishing.

## Figures and Tables

**Table 1:** XRD parameters of the cerium-based abrasive calcined at 400-1050 °C.

T/°C	CeO_2_ (111) 2θ/°	CeO_2_ (111) FWHM/°	LaF_3_ (111) 2θ/°	LaOF (011) 2θ/°	*a*/Å	Scherrer Grain Size/nm	LaF_3_ (111)/CeO_2_ (111) %	LaOF (011)/CeO_2_ (111) %
400	28.511	0.591	27.918		5.412(2)	13.71	14.2	
500	28.572	0.308	27.817		5.413(1)	26.32	12.6	
600	28.614	0.322	27.898		5.409(1)	25.18	11.4	
700	28.675	0.158	27.838		5.404(1)	51.32	8.6	
750	28.714	0.112	27.893		5.401(2)	72.40	5.3	
800	28.714	0.119	27.796		5.401(2)	68.14	4.1	
850	28.676	0.101	27.738		5.403(1)	80.28	1.9	
900	28.656	0.093		26.671	5.405(1)	87.18		1.5
950	28.660	0.104		26.657	5.404(1)	77.96		2.2
1000	28.701	0.111		26.975	5.402(2)	73.05		1.8
1050	28.682	0.110		26.892	5.403(2)	73.71		4.5

**Table 2:** Structural parameters and phase fractions extracted from the Rietveld refinements for the cerium-based abrasive calcined at 400–1050 °C.

Sample	Phase	Space Group	Lattice Parameters (Å)		Phase
			*a*	*c*	Fraction
Calcined at 400 °C	CeO_2_	*Fm*-3*m*	5.4216(2)		90.24(18)%
R_p_ = 2.47%	LaF_3_	*P*-3*c1*	7.1408(6)	7.297(1)	9.76(18)%
R_wp_ = 3.69%					
GOF = 2.04					
Calcined at 800 °C	CeO_2_	*Fm*-3*m*	5.4112(1)		93.05(19)%
R_p_ = 3.09%	LaF_3_	*P*-3*c1*	7.1804(7)	7.346(1)	6.95(19)%
R_wp_ = 5.28%					
GOF = 2.88					
Calcined at 900 °C	CeO_2_	*Fm*-3*m*	5.4112 (1)		98.61(18)%
R_p_ = 3.22%	LaOF	*P*4*/nmm*	4.104(3)	5.829(8)	1.39(18)%
R_wp_ = 5.92%					
GOF = 3.28					
Calcined at 1050 °C	CeO_2_	*Fm*-3*m*	5.4114(1)		95.47(15)%
R_p_ = 3.14%	LaOF	*P*4*/nmm*	4.0784(5)	5.802(1)	4.53(15)%
R_wp_ = 5.60%					
GOF = 3.15					

**Table 3:** XPS binding energy and peak area of individual peak of Ce 3d for the samples prepared at 800 and 900 °C.

		Ce 3d_5/2_					Ce 3d_3/2_					
Peak Assignment		$v_{0}$	$v^{\prime }$	$v_{1}$	$v^{\prime \prime }$	$v^{\prime \prime \prime }$	$u_{0}$	$u^{\prime }$	$u_{1}$	$u^{\prime \prime }$	$u^{\prime \prime \prime }$	
		Ce^3+^	Ce^4+^	Ce^3+^	Ce^4+^	Ce^4+^	Ce^3+^	Ce^4+^	Ce^3+^	Ce^4+^	Ce^4+^	
Temperature 800 °C	Binding energy (eV)	881.8	884.1	886.6	889.7	898.7	900.3	902.6	905.5	908.9	917.7	[Ce^3+^] (%)
	Peak area (%)	5.58	8.96	5.74	11.34	17.24	11.12	12.45	2.69	8.57	16.30	25.13
900 °C	Binding energy (eV)	881.3	883.6	886.4	889.3	897.9	900.0	901.3	904.4	907.9	916.9	[Ce^3+^] (%)
	Peak area (%)	12.17	14.01	5.89	11.08	16.67	3.54	15.79	1.13	4.92	14.79	22.73

## Data Availability

Not applicable.
